# Genetically predicted plasma levels of amino acids and metabolic dysfunction-associated fatty liver disease risk: a Mendelian randomization study

**DOI:** 10.1186/s12916-023-03185-y

**Published:** 2023-11-28

**Authors:** Jian Zhao, Jing Zeng, Cairong Zhu, Xuechao Li, Dong Liu, Jun Zhang, Fei Li, Giovanni Targher, Jian-Gao Fan

**Affiliations:** 1 The Ministry of Education and Shanghai Key Laboratory of Children’s Environmental Health, Xinhua Hospital, Shanghai Jiao Tong University School of Medicine, No. 1665, Kongjiang Road, Yangpu District, Shanghai, 200092 China; 2 Department of Maternal and Child Health, School of Public Health, Shanghai Jiao Tong University, Shanghai, China; 3 MRC Integrative Epidemiology Unit, University of Bristol, Bristol, UK; 4 Department of Gastroenterology, Xinhua Hospital, Shanghai Jiao Tong University School of Medicine, No. 1665, Kongjiang Road, Yangpu District, Shanghai, 200092 China; 5 Department of Epidemiology and Biostatistics, West China School of Public Health and West China Fourth Hospital, Sichuan University, Chengdu, China; 6 Department of Developmental and Behavioral Pediatric & Child Primary Care, Brain and Behavioral Research Unit of Shanghai Institute for Pediatric Research, Xinhua Hospital, Shanghai Jiao Tong University School of Medicine, Shanghai, China; 7 Department of Medicine, University of Verona, Verona, Italy; 8 Metabolic Diseases Research Unit, IRCCS Ospedale Sacro Cuore - Don Calabria, Negrar di Valpolicella, Italy; 9 Shanghai Key Lab of Pediatric Gastroenterology and Nutrition, Shanghai, China

**Keywords:** Amino acids, Metabolomics, MAFLD, Mendelian randomization, Causality

## Abstract

**Background:**

Emerging metabolomics-based studies suggested links between amino acid metabolism and metabolic dysfunction-associated fatty liver disease (MAFLD) risk; however, whether there exists an aetiological role of amino acid metabolism in MAFLD development remains unknown. The aim of the present study was to assess the causal relationship between circulating levels of amino acids and MAFLD risk.

**Methods:**

We conducted a two-sample Mendelian randomization (MR) analysis using summary-level data from genome-wide association studies (GWAS) to evaluate the causal relationship between genetically predicted circulating levels of amino acids and the risk of MAFLD. In the discovery MR analysis, we used data from the largest MAFLD GWAS (8434 cases and 770,180 controls), while in the replication MR analysis, we used data from a GWAS on MAFLD (1483 cases and 17,781 controls) where MAFLD cases were diagnosed using liver biopsy. We used Wald ratios or inverse variance-weighted (IVW) methods in the MR main analysis and weighted median and MR-Egger regression analyses in sensitivity analyses. Furthermore, we performed a conservative MR analysis by restricting genetic instruments to those directly involved in amino acid metabolism pathways.

**Results:**

We found that genetically predicted higher alanine (OR = 1.43, 95% CI 1.13–1.81) and lower glutamine (OR = 0.83, 95% CI 0.73–0.96) levels were associated with a higher risk of developing MAFLD based on the results from the MR main and conservative analysis. The results from MR sensitivity analyses and complementary analysis using liver proton density fat fraction as a continuous outcome proxying for MAFLD supported the main findings.

**Conclusions:**

Novel causal metabolites related to MAFLD development were uncovered through MR analysis, suggesting future potential for evaluating these metabolites as targets for MAFLD prevention or treatment.

**Supplementary Information:**

The online version contains supplementary material available at 10.1186/s12916-023-03185-y.

## Background

Non-alcoholic fatty liver disease (NAFLD) is one of the most prevalent chronic liver diseases, affecting up to ~ 30% of the general population globally [1]. NAFLD has been also predicted to become the most frequent indication for liver transplantation in Western countries by 2030 [2]. NAFLD is a progressive disease characterized by the accumulation of lipid droplets within hepatocytes in the absence of excessive alcohol consumption and defined by the presence of at least 5% hepatic steatosis [3]. This condition has been consistently reported to be associated with important cardiometabolic comorbidities, including obesity, type 2 diabetes mellitus, cardiovascular disease and stroke [4, 5]. In 2020, an international consensus panel of experts proposed a new definition, metabolic dysfunction-associated fatty liver disease (MAFLD), to replace NAFLD, as MAFLD is more comprehensive and independent of other liver diseases [6, 7]. To date, although substantial efforts have been put forth to prevent or treat MAFLD, there are no effective preventions or therapeutic treatments for MAFLD.

Emerging metabolomics-based studies have provided insights into the mechanisms underlying the development and progression of MAFLD [8, 9]. Identifying pathogenic molecules of MAFLD development is essential for improving aetiological understanding and developing novel therapeutic targets for early intervention of this common and burdensome liver disease. It is known that abnormal lipid and glucose metabolism exert putative roles in the pathogenesis of MAFLD [10], whereas recent studies suggested that amino acid metabolism might also contribute to the pathogenesis of MAFLD [11, 12]. For example, lower glycine was reported to be associated with a higher prevalence of MAFLD [13]. Increased levels of aromatic amino acids (AAAs) (e.g. tyrosine and phenylalanine) were found to be associated with increased risk of liver diseases [14]. Increased levels of branched-chain amino acids (BCAAs), including leucine, isoleucine and valine, have also been reported during the progression of MAFLD [15]. In addition, a recent Mendelian randomization study found a causal effect of MAFLD on blood tyrosine levels [16]. Most previous studies have focused on the profiling of amino acids or altered amino acid metabolism in individuals with MAFLD, compared to those without MAFLD, for the discovery of non-invasive diagnostic biomarkers. Metabolism of amino acids including BCAAs, alanine, glutamine and tyrosine has been reported to be impacted by MAFLD [15, 17]. This implies that altered metabolism of amino acids might be a consequence of MAFLD rather than a causal risk factor for MAFLD. Thus, whether there exists an aetiological role of amino acid metabolism in MAFLD development (i.e. a causal effect of circulating levels of amino acids on MAFLD risk) remains currently unknown.

**Fig. 1:**
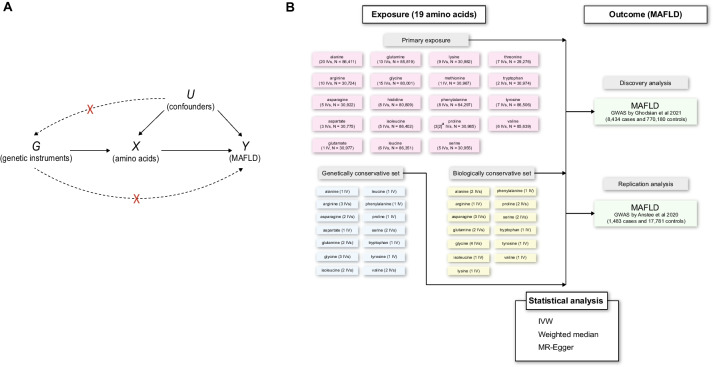
Schematic overview of the study design and MR analysis. a. rs3970551 was absent from the IV set in the replication MAFLD GWAS by Anstee et al. due to non-available proxy SNPs being identified. **A** The causal diagram of the relationship between circulating amino acids and MAFLD investigated in the two-sample MR analysis. **B** The data source of amino acids and MAFLD and relevant information on genetic instrumental variables selected as well as statistical analysis methods used in the study. GWAS, genome-wide association study; IV, instrumental variable; IVW, inverse variance weighted; MAFLD, metabolic dysfunction-associated fatty liver disease; MR, Mendelian randomization

Mendelian randomization (MR) is a causal inference approach using germline genetic variants as instrumental variables (IVs), which could largely minimize the risk of bias due to residual confounding or reverse causation [18, 19]. In this study, we implement two-sample MR analyses to systematically assess the causal effects of genetically predicted circulating levels of amino acids on risk of MAFLD using summary data from both the latest and largest genome-wide association studies (GWASs) of human metabolites and two independent GWASs of MAFLD (Fig. 1A).

## Methods

### Data sources

#### Exposure measure: amino acids

Summary data for genetic associations with amino acids were retrieved from a recently conducted cross-platform GWAS of 174 metabolites that included up to 86,507 participants (for individual metabolites sample sizes varied from 8569 to 86,507) [20]. Genome-wide association results were meta-analysed in three cohort studies (i.e. the Fenland, EPIC-Norfolk and INTERVAL studies) followed by a further meta-analysis with publicly available GWAS summary data from two studies [21, 22]. Of the 174 plasma metabolites investigated in the GWAS, 20 circulating levels of amino acids (alanine, arginine, asparagine, aspartate, cysteine, glutamate, glutamine, glycine, histidine, isoleucine, leucine, lysine, methionine, phenylalanine, proline, serine, threonine, tryptophan, tyrosine and valine) were included. We calculated the single nucleotide polymorphism (SNP)-specific genetic associations with amino acids and their corresponding standard errors based on the original information, including the *z*-score, sample size and minor allele frequency (MAF), reported in the GWAS of metabolites using the following formula $\widehat{b}=z/\sqrt{2p(1-p)(n+{z}^{2})}$ and $SE=1/\sqrt{2p(1-p)(n+{z}^{2})}$ [23], where *b* is the SNP-specific genetic association, *z* is the *z*-score, *p* is the MAF of the SNP, *n* is the sample size and SE is the standard error of the genetic association.

#### Outcome measure: MAFLD

In our study, we used MAFLD instead of NAFLD, as it has been proposed as a more appropriate and comprehensive term to define the liver disease associated with known metabolic dysfunction according to the recent consensus statement by an international panel of experts [6]. Two independent datasets on the outcome measure (i.e. MAFLD) were retrieved from recently conducted GWASs of MAFLD [24, 25]. Data for discovery analysis were obtained from the largest MAFLD GWAS meta-analysis conducted in four cohorts of European ancestry: the Electronic Medical Records and Genomics (eMERGE) network, the UK Biobank (UKB), the Estonian Biobank (EstBB) and the FinnGen [24]. In the GWAS meta-analysis on MAFLD, two GWASs of MAFLD were firstly conducted in the UKB and EstBB cohorts and then combined with the results from two publicly available MAFLD GWASs (eMERGE and FinnGen). As a result, 8434 MAFLD cases were identified by electronic health records (EHR), and 770,180 controls were included in the GWAS meta-analysis (Fig. 1B). Furthermore, for the replication analysis, data were retrieved from a large GWAS of MAFLD in individuals of European ancestry (1483 cases and 17,781 controls), where MAFLD cases were diagnosed using liver biopsy (i.e. the gold standard method for diagnosing MAFLD) [25] (Fig. 1B).

### Genetic instruments selection and data harmonization

Based on the GWAS summary data on cross-platform measured metabolites, 112 genetic variants, which were associated with at least one of 20 circulating amino acids at a metabolome-adjusted genome-wide significance level (*p* < 5 × 10^−8^/102 = 4.9 × 10^−10^), were selected as candidate IVs. In the present study, a stringent linkage disequilibrium (LD) clumping threshold (*r*^2^ < 0.01 and window = 10 Mb) for genetic instruments selection was applied using the “clump_data” function in the TwoSampleMR R package. A total of 111 SNPs (excluding rs61937878) were retained after LD clumping.

Each genetic instrument was looked up in two MAFLD GWASs (for discovery and replication analysis) for SNP-MAFLD associations. SNPs that are in high LD with genetic instruments (*r*^2^ > 0.8 and window = 500 Mb) were identified to proxy the absent variants in the discovery and replication MAFLD datasets (14 and 6 proxies were identified, respectively). Detailed information on the proxy SNPs can be found in the Additional file 1: Table S1. Due to the absence of proxy SNPs available in the summary data of MAFLD GWAS, one SNP (rs142714816, the unique instrument for cysteine) was excluded from the discovery analysis, and two SNPs (rs142714816 and rs3970551) were removed from the replication analysis.

A data harmonization procedure was performed to merge SNP-amino acid and SNP-MAFLD associations using the “harmonise_data” function in the TwoSampleMR R package [26]. Two palindromic SNPs (rs2422358 and rs1935) were removed from further analysis. As a result, a total of 108 and 107 eligible SNPs used as instrumental variables for 19 circulating amino acids were included in the discovery and replication MR analysis, respectively.

### Statistical analysis

In both discovery and replication stages, we used Wald ratios (for glutamate and methionine because only one SNP was available for each of these two amino acids), and the fixed-effects inverse variance-weighted (IVW) method when there were three or fewer genetic instruments available or the random-effects IVW method otherwise for all other amino acids as the MR main analysis method, to estimate the causal effect of genetically predicted circulating levels of amino acids on risk of having MAFLD. To increase the statistical power and precision of the causal estimates, a fixed-effect meta-analysis was performed to combine the causal estimates in both the discovery and replication stages using the meta R package [27].

Additionally, for certain amino acids that have four or more genetic instruments, we performed several sensitivity analyses, including weighted median and MR-Egger regression analysis to test the consistency of the causal estimates under the different assumptions and to detect possible pleiotropy. Unlike the IVW method that assumes all the SNPs are valid IVs [28], the MR-Egger regression could generate a consistent estimate in the presence of invalid genetic instruments, as long as the Instrument Strength Independent of Direct Effect (InSIDE) assumption holds [29]. The weighted median method assumes that more than half of the genetic instruments are valid and is a robust approach to outliers [30]. To minimize the risk of violating the MR assumptions, we identified genetic variants associated with potential confounders such as body mass index (BMI), waist-to-hip ratio and whole-body fat mass by searching the PhenoScanner database [31] and compared the MR analysis results after excluding these SNPs with our MR main analysis results.

To assess the strength of the selected genetic instruments in MR analysis, we calculated the *F* statistics for each genetic instrument, which are generally considered strong when greater than 10 [32]. We used Cochrane’s *Q* statistic to examine the heterogeneity between SNP-specific causal estimates. Substantial heterogeneity between SNP-specific causal estimates could be indicative of horizontal pleiotropy. As the discovery data involved four separate cohorts with quite different proportions of MAFLD and considering the potential false-negative misclassification for MAFLD, we also conducted MR analysis separately for each of the individual cohorts for which we had access to the GWAS summary data. We assessed the between-cohort heterogeneity of causal effect estimates using Cochrane’s *Q* statistic.

Furthermore, to minimize the risk of bias due to horizontal pleiotropy, we also performed a conservative MR analysis by restricting genetic instruments to those directly involved in amino acid metabolism pathways, as described elsewhere [33]. Two sets of genetic variants, namely biologically and genetically prioritized conservative SNPs, were used as instrumental variables in conservative MR analysis to estimate the causal effects using the Wald ratios or fixed-effect IVW method as appropriate.

To explore the potential that altered metabolism of amino acids might be a consequence of MAFLD rather than an aetiological factor for MAFLD, we conducted a reverse direction MR analysis considering MAFLD as the exposure and the levels of amino acids as the outcome. Four SNPs, rs3747207 (*PNPLA3*), rs429358 (*APOE*), rs73001065 (*TM6SF2*) and rs28601761 (*TRIB1*), reported as genome-wide significant loci for MAFLD [24] were chosen as genetic instruments for MAFLD. The fixed-effects IVW method, along with the weighted median and weighted mode methods, were used in the MR analysis.

Finally, to validate the findings in the primary MR analysis, we repeated MR analysis using liver proton density fat fraction (PDFF) derived from magnetic resonance imaging (MRI) of 36,116 individuals of European ancestry from the UKB as a continuous outcome. To maintain consistency with the primary MR analysis, we employed the Wald ratios method for glutamate and methionine, as they each had only one SNP serving as an instrumental variable. For tryptophan, proline and aspartate, we applied the fixed-effects IVW method. Meanwhile, for the remaining amino acids, we utilized the multiplicative random-effects IVW method.

Given that a total of 20 amino acids were investigated in the present study, after a multiple testing Bonferroni correction, an estimate with a *p*-value < 0.0025 (*p* = 0.05/20) was considered as strong evidence for causal effects, whereas a *p*-value between 0.0025 and 0.05 indicated a suggestive causal effect. All statistical analyses were undertaken with R version 4.0.2 (R Foundation for Statistical Computing, Vienna, Austria).

## Results

### Characteristics of the included studies and the selected SNPs

Genetic variants instrumenting for amino acids in our study were obtained from a meta-analysis of metabolite GWAS using data from up to 23 cohorts included in three previous GWASs [22, 34, 35] and three independent studies (the Fenland cohort [36], EPIC-Norfolk Study [37] and INTERVAL trial [38]) (Table 1). The average participant age of the included studies ranged from 43.5 to 59.8 years old [20]. Approximately 50.4 to 53.9% of the study participants were women, except for the GWAS conducted by Shin et al. [22] where only 16.5% of participants were women. A large-scale meta-analysis of MAFLD GWASs in four studies of European ancestry (the eMERGE, FinnGen, UKB and EstBB cohorts) included 8434 MAFLD cases and 770,180 controls and was used for discovery MR analysis [24]. Another independent MAFLD GWAS used for the replication analysis included 1483 MAFLD cases diagnosed with liver biopsy, and 47.3% of these participants were women.

**Table 1:** Characteristics of summary level data used in the MR analysis

**Studies**	**Sample size (*n*)**		**Female %**	**Mean age (SD)**	**Metabolomics platform**		**Ancestry**
**Amino acids (meta-analysis of metabolites GWAS)**							
Fenland study	9736		53.5%	48.4 (7.4)	Biocrates p180 Kit		European
EPIC-Norfolk study	5841		53.3%	59.8 (9.0)	Metabolon HD4		
INTERVAL trial	40,818		50.4%	43.5 (14.2)	Serum NMR platform (Nightingale) and Metabolon HD4		
GWAS by Shin et al. (2014)	7824		16.5%	57.1 (11.4)	Metabolon HD1		
GWAS by Draisma et al. (2015)	7478		53.5%	48.7 (12.7)	Biocrates p150 Kit		
GWAS by Kettunen et al. (2016)	24,925		53.9%	46.3 (9.7)	Serum NMR platform (Nightingale)		
**Studies**	**Cases**	**Controls**			**Inclusion criteria**	**Exclusion criteria**	
**MAFLD (studies for discovery analysis)**							
eMERGE	Adults: 710/paediatrics (≤ 21 years old): 396	Adults: 7725/paediatrics: 846	Adults: 54.8%/paediatrics: 44.2%	Adults: 63.5 (16.9)/paediatrics: 13.1 (5.4)	ICD9: 571.5, 571.8 and 571.9; ICD10: K75.81, K76.0 and K76.9	Alcohol dependence, alcoholic liver disease, alpha-1 antitrypsin deficiency, Alagille syndrome, liver transplant, cystic fibrosis, hepatitis, abetalipoproteinemia, LCAT (lecithin-cholesterol acyltransferase) deficiency, lipodystrophy, disorders of copper metabolism Reye’s syndrome, inborn errors of metabolism, HELLP (hemolysis, elevated liver enzymes and low platelets) syndrome, starvation and acute fatty liver	European
UK Biobank	2558	395,241	NA	NA	ICD10: K74.0, K74.2, K75.8, K76.0 and K76.9		
Estonian Biobank	4119	190,120	NA	NA	ICD10: K74.0, K74.2, K75.8, K76.0 and K76.9		
FinnGen study (data freeze 4)	651	176,248	55.5%^a^	52.1^a^	ICD10: K76.0	NA	
**MAFLD (studies for replication analysis)**							
GWAS by Anstee et al. (2020)	1483	17,781	47.3%^a^	50.1 (13.0)^a^	Liver biopsy	Excess alcohol intake (alcohol intake < 20 g/day for females; < 30 g/day for males), chronic viral hepatitis (hepatitis B and hepatitis C), autoimmune liver diseases, hereditary hemochromatosis, α1-antitrypsin deficiency, Wilson’s disease and drug-induced liver injury	European

^a^The percentage of women or mean age was calculated in the MAFLD case group only

The characteristics of the selected SNPs instrumenting for amino acids are presented in Additional file 1: Table S2. A total of 133 and 134 genetic variants were used as IVs, ranging from 1 IV (for glutamate and methionine) to 20 IVs (for alanine), to estimate the causal effects of 19 amino acids on MAFLD in discovery and replication MR analysis, respectively. The *F*-statistics of genetic variants instrumenting for 19 amino acids ranged from 38.7 to 7504.1 (Additional file 1: Table S2), suggesting a low risk of weak instrument bias. Associations between the genetic instruments and the outcome MAFLD can be found in Additional file 1: Table S3. Proportions of variation in amino acids explained by genetic instruments ranged from 0.13% (glutamate) to 10.38% (glycine) (Additional file 1: Table S4). Cochrane’s *Q* statistic indicated that there was no significant heterogeneity between SNP-specific causal estimates for arginine, aspartate, phenylalanine, proline and tryptophan in the discovery MR analysis (Additional file 1: Table S5).

### MR main analysis results

**Fig. 2:**
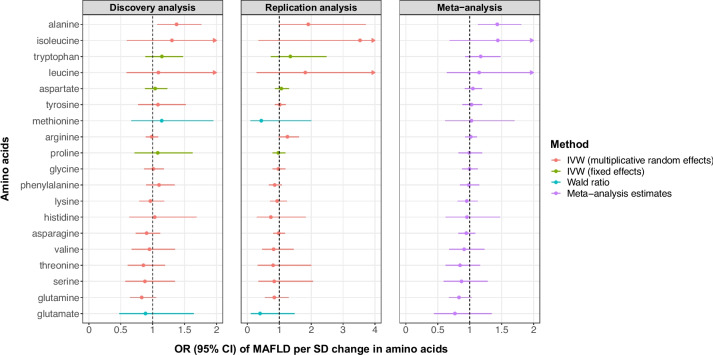
MR main analysis results of the causal effects of genetically predicted circulating levels of amino acids on MAFLD risk. Wald ratios method was used for glutamate and methionine; the fixed-effects IVW method was used for tryptophan, aspartate and proline; and the multiplicative random-effects IVW method was used for the remaining amino acids. Meta-analysis was used to combine the causal effect estimates derived in the discovery and replication analysis. CI, confidence intervals; IVW, inverse variance weighted; MAFLD, metabolic dysfunction-associated fatty liver disease; MR, Mendelian randomization; OR, odds ratio; SD, standard deviation

Of the 19 amino acids examined, genetically predicted higher circulating alanine levels were causally associated with an increased risk of MAFLD in both discovery and meta-analyses. The odds ratio (OR) of MAFLD was 1.43 (95% CI 1.13–1.81; *p* = 0.002) per 1-SD increment in alanine levels, after combining causal effect estimates from discovery (OR = 1.37, 95% CI 1.07–1.76; *p* = 0.012) and replication (OR = 1.91, 95% CI 0.98–3.71; *p* = 0.056) MR analyses (Fig. 2). There was little evidence for a causal association between circulating levels of the remaining amino acids and MAFLD risk. Causal effect estimates from the replication MR analysis were broadly consistent with those from the discovery analysis, except for methionine, which had discrepant directions of effect but low precisions.

### MR sensitivity analyses results

MR sensitivity analyses, including weighted median and MR-Egger regression analyses, were conducted in 14 amino acids that had at least 4 SNPs as genetic instruments (Additional file 1: Table S6). A broad consistency was observed when comparing the results from sensitivity analyses with those from the main analysis presented above, except for several amino acids which had very low precisions in replication MR-Egger regression analysis. Reasons for the low precisions of estimates included smaller sample size of data used in replication analysis and homogeneous SNP-amino acid associations [39]. Of note, meta-analysed causal effect estimates derived from weighted median analysis, which is statistically more robust compared to MR-Egger regression, supported potential causal effects of both alanine (OR = 1.58, 95% CI 1.22, 2.04; *p* < 0.001) and glutamine (OR = 0.81, 95% CI 0.70, 0.93; *p* = 0.004) on MAFLD risk.

We identified 22 genetic variants associated with BMI, waist-to-hip ratio and whole body fat mass after searching the PhenoScanner database, and then we performed MR analysis after excluding these potentially pleiotropic SNPs (Additional file 1: Table S7). We found that alanine, glycine and threonine appeared to have a positive causal effect on MAFLD risk, and glutamine and leucine were inversely associated with MAFLD risk (Additional file 2: Fig. S1).

We were able to access GWAS summary data on MAFLD in three of four individual cohorts (i.e. eMERGE, FinnGen and UKB) included in the GWAS done by Ghodsian et al. [24]. We conducted MR analysis for each individual cohort separately and calculated the between-cohort heterogeneity of causal effect estimates using Cochrane’s *Q* statistic (Additional file 2: Fig. S2 and Additional file 1: Table S8), which suggested little heterogeneity of causal estimates between cohorts.

### Conservative MR analysis results

**Fig. 3:**
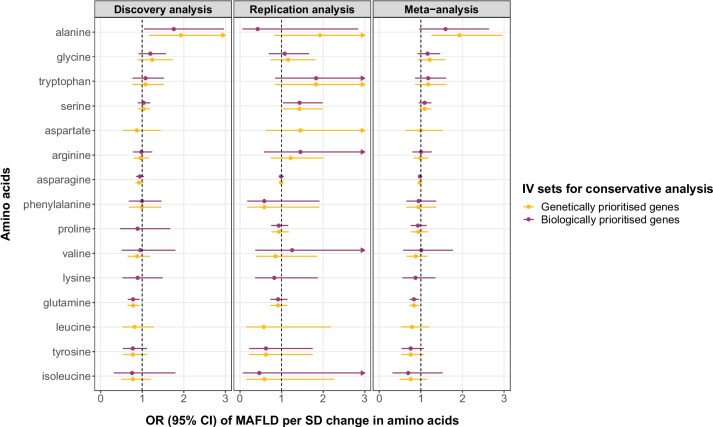
MR conservative analysis results using genetically and biologically prioritized variants as instrumental variables. The genetic instruments we chose were prioritized in the original GWAS on metabolites using two approaches, namely a hypothesis-free genetic approach and a biological knowledge-based approach [20], which suggested likely causal genes for the amino acids. CI, confidence intervals; IV, instrumental variable; MAFLD, metabolic dysfunction-associated fatty liver disease; OR, odds ratio; SD, standard deviation

By restricting genetic instruments for amino acids to SNPs that were biologically or genetically prioritized in previous published GWAS of metabolites [20], we performed a conservative MR analysis to achieve a more reliable causal inference. We were unable to investigate histidine, threonine, methionine and glutamate as genetic variants instrumenting for these amino acids were not on the list of biologically or genetically prioritized genes nor directly involved in relevant metabolism pathways. The results from the conservative MR analysis confirmed a causal role of alanine (OR = 1.93, 95% CI 1.26, 2.96, *p* = 0.003 for genetically prioritized IVs) and glutamine (OR = 0.83, 95% CI 0.73, 0.96, *p* = 0.009 for both biologically and genetically prioritized IVs) on the risk of MAFLD (Fig. 3).

### Causal effects of MAFLD on amino acids

**Fig. 4:**
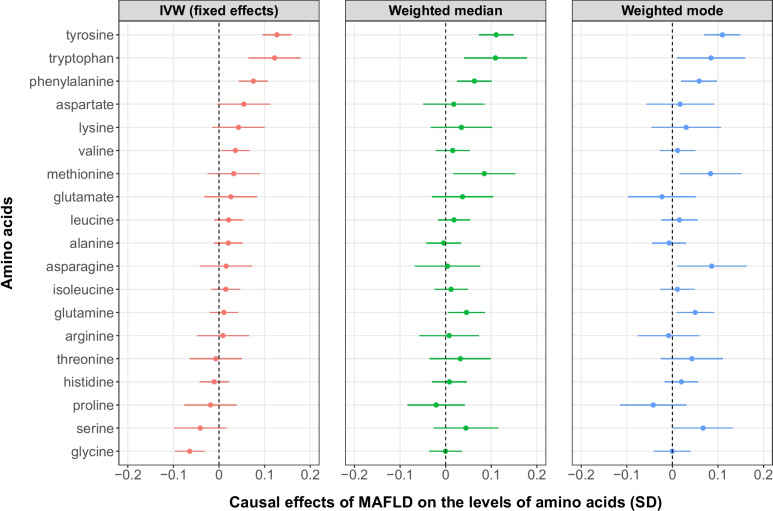
Causal effects of MAFLD on the levels of amino acids. Four SNPs, rs3747207 (*PNPLA3*), rs429358 (*APOE*), rs73001065 (*TM6SF2*) and rs28601761 (*TRIB1*), were used as instrumental variables for MAFLD to infer the causal effects of genetically predicted MAFLD on the levels of amino acids. *APOE*, apolipoprotein E; *PNPLA3*, Patatin-like phospholipase domain containing 3; *TM6SF2*, transmembrane 6 superfamily member 2; *TRIB1*, Tribbles pseudokinase 1; MAFLD, metabolic dysfunction-associated fatty liver disease; SD, standard deviation

In the reverse direction MR analysis using MAFLD as the exposure and the levels of amino acids as the outcome, we found that individuals with higher genetic liability to MAFLD were more likely to have higher levels of AAAs (including tyrosine, tryptophan and phenylalanine) and valine and lower levels of glycine, among which the causal evidence on all the AAAs were strengthened by the results from the weighted median and weighted mode analyses (Fig. 4).

### Validation analysis using PDFF as an outcome

**Fig. 5:**
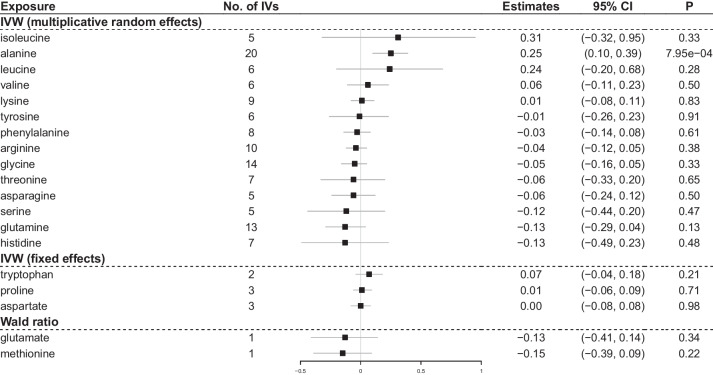
PDFF difference (SD) per SD change in amino acids. PDFF measured in the UKB was used as a continuous outcome proxying for MAFLD to validate the primary MR analysis results. CI, confidence intervals; IV, instrumental variable; IVW, inverse variance weighted; MR, Mendelian randomization; P, *p* value; PDFF, proton density fat fraction; SD, standard deviation; UKB, UK Biobank

When using the PDFF measured in the UKB as a continuous outcome to validate our primary MR analysis findings, we observed that genetically predicted higher alanine was positively associated with higher PDFF (Fig. 5), which was consistent with the positive causal association between alanine and MAFLD risk revealed in our MR main analysis.

## Discussion

In this MR study, we provided novel evidence for a causal role of genetically predicted circulating levels of alanine and glutamine in the development of MAFLD. Specifically, genetically predicted higher alanine and lower glutamine were associated with a higher risk of developing MAFLD. To the best of our knowledge, it is the first study systematically assessing the causal relationships between levels of plasma amino acids and the development of MAFLD using multi-omics (i.e. genomic and metabolomic) data from large-scale human studies (in up to 778,614 individuals).

Previous observational studies mainly focused on the profiling of amino acids or altered amino acid metabolism in individuals with MAFLD compared with those without MAFLD. Metabolism of amino acids including BCAAs (i.e. leucine, isoleucine and valine), alanine, glutamine and tyrosine has been reported to be impacted by MAFLD [15, 17]. These findings were beneficial to identifying diagnostic biomarkers of MAFLD, whereas they were not capable of providing causal evidence for aetiological biomarkers of MAFLD development. Thus, our findings provide novel insights into the causal mechanism between altered amino acid metabolism and MAFLD development.

Glutamate is one of the major substrates for the synthesis of glutathione, which is a tripeptide consisting of glutamate, cysteine and glycine and protects tissues from free radical injury via detoxification of active species and/or repair of injury. Since glutamate is poorly transported into cells and glutamine can be efficiently transported across the cell membrane and deaminated in the mitochondria to produce glutamate and NH3, plasma glutamine is thus important for the generation of intracellular glutamate and consequently glutathione. Experimentally, it has been reported a potential causal role of glutamine administration in decreasing liver injury and mortality in animal studies [40–42]. However, there are sparce human studies on the effect of glutamine administration on liver function and its related biomarkers. The present study, from a genetic perspective, provides causal evidence for a protective causal effect of higher circulating glutamine levels on the development of MAFLD. Further, in our MR conservative analysis, we found that only the *GLS-2* (rs2657879) genetic variant predicted glutamine exerting a causal effect on MAFLD risk, compared with another variant (*GLS*, rs7587672) instrumenting for glutamine. Our results were partly supported by findings from a previous study, where the authors found that reducing glutamine metabolism (loss-of-function of *GLS2)* in the liver resulted in decreased severity of hyperglycaemia (increased plasma levels of glutamine and reduced levels of fasting glucose) [43].

Alanine is the primary amino acid involved in hepatic gluconeogenesis. Abnormal levels of alanine typically signal a disruption in the alanine-glucose cycle, which can subsequently impact the tricarboxylic acid (TCA) and urea cycles [44, 45]. Elevated alanine concentrations in MAFLD have been observed in multiple latest metabolomics studies [46], which was consistent with an emerging hypothesis of dysregulated TCA and urea cycles in MAFLD [47, 48]. Recently, in the Young Finns Study that examined prospective associations between baseline metabolite levels and the future risk of MAFLD, plasma alanine levels were also found to be positively associated with the future onset of MAFLD [49]. In our study, the results from MR analysis confirmed the positive causal effect of circulating levels of alanine on MAFLD risk.

Among other amino acids, higher levels of BCAAs (including leucine, isoleucine and valine) and AAAs (including phenylalanine, tryptophan and tyrosine) have been reportedly linked with MAFLD [50–52]; however, to our knowledge, we identified only one study in which the prospective associations between baseline concentrations of amino acids and the risk of developing MAFLD during the 10-year follow-up were examined [49]. Interestingly, in our study, contrary to the positive associations revealed in the above-mentioned studies, we find little evidence to support a causal effect of both BCAAs and AAAs on MAFLD development. One reason for these discordant results might be reverse causation or confounding bias that cannot be ruled out in previous observational studies. For example, in the prospective Young Finns Study, increased plasma tyrosine levels were associated with a higher 10-year risk for fatty liver when first adjusted for sex and age, whereas after adjusting for additional baseline confounders, such as waist circumference, alcohol intake, smoking and leisure-time physical activity, this association attenuated and became statistically non-significant [49]. Further, in a recent MR study investigating the causal effect of MAFLD on consequent blood metabolites, MAFLD was found to have a positive impact on plasma tyrosine levels [16]. In our reverse direction MR analysis, the causal effect direction from MAFLD to the levels of all the AAAs was also confirmed. Taken together with our results, it seems more plausible to consider altered AAA metabolism as a response to the presence of MAFLD rather than an aetiological factor for MAFLD development.

Our study has several strengths. Firstly, this is the first and largest study systematically investigating the causal effects of human circulating amino acids on MAFLD risk, utilizing multi-omics data. Secondly, we leveraged data from an independent GWAS of MAFLD to validate our findings in the discovery population, and combined causal effect estimates from both datasets using meta-analysis to increase statistical power and estimate precision. Thirdly, the conservative MR analysis that was less susceptible to horizontal pleiotropy using genetically and biologically prioritized SNPs as instrumental variables confirmed the findings from our MR main analysis. Finally, our results can be generalized to European ancestry as samples span the entire Europe.

We acknowledge some important limitations of our study. Firstly, our study was limited to individuals of European ancestry due to data availability; thus, generalizability to other ethnic populations needs to be further investigated. Secondly, although summary data from the largest histology-based MAFLD GWAS was used to replicate our findings, the results derived from discovery analysis were based on EHR data where the diagnosis of MAFLD may be biased by misclassification of cases and controls due to using hospital records (i.e. ICD-9 and ICD-10 codes). Therefore, future replications in larger cohorts of participants with MAFLD diagnosed with the gold standard (i.e. liver biopsy) are warranted.

## Conclusions

In conclusion, novel causal biomarkers including alanine and glutamine of MAFLD development were revealed in our study with integrating genomic and metabolomic data. Future research on how healthy diets or lifestyle modifications affect these newly identified causal metabolites, in order to design better preventive or intervention strategies aimed at reducing the burden of MAFLD, is warranted.

### Supplementary Information

**Additional file 1:** **Table S1.** Proxy SNPs of genetic instruments identified in the MAFLD summary data. **Table S2.** Characteristics of SNPs instrumenting for amino acids in the MR analysis. **Table S3.** Genetic associations between genetic instrumental variables and MAFLD in two GWAS data used for discovery and replication analysis. **Table S4.** Proportion of variation in amino acids explained by genetic instruments. **Table S5.** Heterogeneity tested between SNP-specific causal estimates in the discovery MR analysis. **Table S6.** MR sensitivity analysis results using weighted median and MR-Egger regression methods. **Table S7.** Genetic variants associated with BMI, waist-to-hip ratio and whole body fat mass by searching the PhenoScanner database. **Table S8.** MR analysis results for each individual cohort and Cochrane’s *Q* statistic.

**Additional file 2: Fig. S1.** MR analysis results after excluding SNPs associated with BMI, waist-to-hip ratio and whole body fat mass after searching the PhenoScanner database. **Fig. S2.** MR analysis results for each individual cohort involved in the discovery data.

## Data Availability

Genetic association estimates for amino acids were obtained by accessing to ‘omicscience’ web (https://omicscience.org). The summary statistics on MAFLD were obtained from GWASs conducted by Ghodsian et al. and Anstee et al., which had been deposited in the GWAS catalogue (https://www.ebi.ac.uk/gwas/).

## Abbreviations

AAAs: Aromatic amino acids
BCAAs: Branched-chain amino acids
EHR: Electronic health records
eMERGE: Electronic Medical Records and Genomics
EstBB: Estonian Biobank
GWASs: Genome-wide association studies
InSIDE: Instrument Strength Independent of Direct Effect
IVs: Instrumental variables
IVW: Inverse variance-weighted
LD: Linkage disequilibrium
MAF: Minor allele frequency
MAFLD: Metabolic dysfunction-associated fatty liver disease
MR: Mendelian randomization
NAFLD: Non-alcoholic fatty liver disease
OR: Odds ratio
SNP: Single nucleotide polymorphisms
TCA: Tricarboxylic acid
UKB: UK Biobank
