# Supplementary material for: Genetically predicted plasma levels of amino acids and metabolic dysfunction-associated fatty liver disease risk: a Mendelian randomization study
Source: BMC Med. 2023 Nov 28;21:469. doi: 10.1186/s12916-023-03185-y (PMC10685523; doi:10.1186/s12916-023-03185-y)
Supplement: Supplementary file 2 — Additional file 2: Fig. S1. MR analysis results after excluding SNPs associated with BMI, waist-to-hip ratio and whole body fat mass after searching the PhenoScanner database. Fig. S2. MR analysis results for each individual cohort involved in the discovery data. [file 12916_2023_3185_MOESM2_ESM.docx]

Additional file 2: Supplementary Figures

**Figure S1** MR analysis results after excluding SNPs associated with BMI, waist-to-hip ratio and whole body fat mass after searching the PhenoScanner database.

**Figure S2** MR analysis results for each individual cohort involved in the discovery data.

**Figure S1** MR analysis results after excluding SNPs associated with BMI, waist-to-hip ratio and whole body fat mass after searching the PhenoScanner database.

**Figure S2** MR analysis results for each individual cohort involved in the discovery data.
